# Development of a heart failure filter for Medline: an objective approach using evidence-based clinical practice guidelines as an alternative to hand searching

**DOI:** 10.1186/1471-2288-11-12

**Published:** 2011-01-28

**Authors:** Raechel A Damarell, Jennifer Tieman, Ruth M Sladek, Patricia M Davidson

**Affiliations:** 1 Department of Palliative and Supportive Services, Flinders University, South Australia, Australia; 2 Research to Practice Group, Flinders University/Flinders Medical Centre, Bedford Park, South Australia, Australia; 3 Centre for Cardiovascular and Chronic Care, Curtin University and University of Technology, Sydney, New South Wales, Australia

## Abstract

**Background:**

Heart failure is a highly debilitating syndrome with a poor prognosis primarily affecting the elderly. Clinicians wanting timely access to heart failure evidence to provide optimal patient care can face many challenges in locating this evidence.

This study developed and validated a search filter of high clinical utility for the retrieval of heart failure articles in OvidSP Medline.

**Methods:**

A Clinical Advisory Group was established to advise study investigators. The study set of 876 relevant articles from four heart failure clinical practice guidelines was divided into three datasets: a Term Identification Set, a Filter Development Set, and a Filter Validation Set. A further validation set (the Cochrane Validation Set) was formed using studies included in Cochrane heart failure systematic reviews. Candidate search terms were identified via word frequency analysis. The filter was developed by creating combinations of terms and recording their performance in retrieving items from the Filter Development Set. The filter's recall was then validated in both the Filter Validation Set and the Cochrane Validation Set. A precision estimate was obtained post-hoc by running the filter in Medline and screening the first 200 retrievals for relevance to heart failure.

**Results:**

The four-term filter achieved a recall of 96.9% in the Filter Development Set; 98.2% in the Filter Validation Set; and 97.8% in the Cochrane Validation Set. Of the first 200 references retrieved by the filter when run in Medline, 150 were deemed relevant and 50 irrelevant. The post-hoc precision estimate was therefore 75%.

**Conclusions:**

This study describes an objective method for developing a validated heart failure filter of high recall performance and then testing its precision post-hoc. Clinical practice guidelines were found to be a feasible alternative to hand searching in creating a gold standard for filter development. Guidelines may be especially appropriate given their clinical utility. A validated heart failure filter is now available to support health professionals seeking reliable and efficient access to the heart failure literature.

## Background

Heart failure is a complex syndrome associated with reduced quality of life and poor prognostic outlook [1-3]. It has been identified as a major ongoing public health concern in developed countries due to an increase in the prevalence of risk factors such as diabetes, obesity, and hypertension [4]. The ageing population also represents a significant concern for health systems in the near future given that the likelihood of developing heart failure rises steeply with age [5,6]. There is growing recognition that heart failure patients have many unmet needs, especially in relation to palliative and end-of-life care [7-11]. Health care policies and clinical practice guidelines have accordingly begun to advocate a palliative care approach within general heart failure care [12-16].

Clinicians caring for individuals with heart failure require effective engagement with the rapidly accumulating research evidence in order to provide optimal patient care but can face many challenges in retrieving literature. The Medline database, produced by the National Library of Medicine, is arguably the premiere index of biomedical journal literature due to its breadth of coverage and ready accessibility through PubMed. The size of this resource, however, can make efficient searching difficult [17]. Medline currently contains over 18 million references with a further 2000 to 4000 added on a weekly basis [18]. This means clinicians searching for literature can be confronted with large numbers of retrieved references which they must sift through to identify clinically useful evidence. Furthermore, optimal search construction is an iterative, time-consuming process which relies on familiarity with a database's native syntactical rules, navigational features, and idiosyncratic controlled vocabulary. As a result, busy clinicians may lack the knowledge, skills and resources to search well [19].

Validated search filters offer a sophisticated solution to the problems inherent in bibliographic database searching by being objectively derived, pre-tested strategies with known levels of performance and reproducibility in the databases for which they were created. Filters are increasingly reported in the literature [20,21] and filter development methodology continues to evolve. As it does, so do efforts to provide end-users with a critical appraisal framework to determine fitness for purpose [22,23]. This study reports on the development of a heart failure filter. In addition, it aims to contribute to the emerging field of filter methodology in three ways: by examining concepts relevant to topic-based filters, as distinct from methodological filters; by trialling an alternative approach to the traditional but costly hand search method of forming a gold standard; and by using a post-hoc screening process to estimate clinical relevance and therefore filter precision outside the development and testing datasets.

To date, most search filters are methodological in their focus, developed to retrieve original studies based on a particular research design such as the randomised controlled trial [24,25]. Topic-based filters, in comparison, allow clinicians to search for articles with a specific topic focus [26-29]. While topic filters are less numerous than their methodological counterparts, they are arguably of equal importance, facilitating access to literature relevant to the topic of interest. Methodological filters are designed to be combined with user queries [30]. However, the overall performance of the combined search reflects the quality of the topic search. Given variability in searching skills, for many searchers quality may only be improved if a rigorously developed methodological search filter is combined with an equally well developed and validated topic filter [26].

The choice of a suitably sized, representative gold standard set is crucial to filter development. Hand searching a range of journal titles has commonly been used. It is, however, a laborious, expensive process and may be prone to subjective bias in terms of journal titles selected and any imposed date range limits [31]. An alternative and possibly more efficient approach has been described which uses the included studies from multiple systematic reviews to form a gold standard [32]. This study takes yet another approach by employing a gold standard created from the included studies of multiple evidence-based clinical practice guidelines. Evidence-based guidelines offer a clinically relevant gold standard and, like systematic reviews, are based on the systematic identification of relevant scientific evidence to safeguard against bias [33]. Unlike systematic reviews, which are restricted to a single, focussed clinical question, guidelines usually employ a multidisciplinary process to make recommendations on a range of issues [34]. Their development processes, holistic approach to a content area, and clinical applicability provide an argument for their suitability as a gold standard for building a filter of high clinical utility. A filter for retrieving sex-specific clinical evidence was recently developed using clinical practice guidelines as one source of articles for the gold standard set [35].

The key difference between the systematic review or guideline approach and the traditional one is that these alternative approaches create a set entirely comprised of relevant references while a hand search produces a closed universe comprised of both relevant and irrelevant references. When all references in a set are relevant, it is only possible to evaluate search performance in terms of recall (or sensitivity). Recall is defined as the proportion of all relevant references correctly retrieved. When references identified as irrelevant are included within the dataset it is possible to measure both specificity and precision in addition to recall. Both specificity and precision are measures of the discriminatory power of a search and provide an indication of its outer limits, or what it will not retrieve. Specificity is the proportion of all irrelevant references correctly excluded while precision is the number of relevant references retrieved as a proportion of the total number of references retrieved. For a busy clinician, precision may be considered an indication of the quality of a search as a high precision search delivers a well-contained set of results closely matched to the user's query. Consequently, less time is required to find the relevant amongst the irrelevant [36]. As the aim of this study was to develop and validate a filter for retrieving clinically relevant references on heart failure, it was vital to incorporate a method for gauging the filter's discriminatory power. This was achieved by a post-hoc relevance screening by a clinician. This may be considered an inversion of the hand search method where clinical screening for relevance takes place prior to filter development.

## Methods

The study design had six phases: construction of the gold standard set; term identification; filter development; filter testing; external validation in an alternative gold standard set; and an estimate of precision which we call 'the post-hoc precision estimate'. All Medline searches were performed using the OvidSP interface between March and May 2010. A Clinical Advisory Group was established to provide ongoing clinical assistance and review during the study.

### Phase 1: Construction of the gold standard set

#### Guideline selection

A literature search for heart failure clinical practice guidelines was conducted using a simple strategy (*heart failure *MeSH or equivalent, exploded to capture narrower concepts, combined with the textword guideline* or a guideline publication type limit). Databases searched were Medline, PubMed, Embase, CINAHL, PsycInfo, and Ageline. Clinical Evidence, UpToDate, and the websites of major guideline development organisations were also consulted. Retrieved guidelines were matched against predetermined criteria developed with the Clinical Advisory Group. To be considered for inclusion, a guideline needed to be: written in English; published no earlier than 2005; intended for use at a national, rather than organisational level; and available online as a PDF file. It needed to be relatively broad in scope, covering the topic from definition and epidemiology through to diagnosis, treatment, self management and end-of-life issues. Furthermore, each guideline needed to meet standards set by the AGREE quality appraisal tool with respect to search and appraisal processes [37]. Several major guidelines were eliminated based on these criteria.

The four guidelines chosen to create the gold standard were those produced by the National Heart Foundation Australia [38]; European Society of Cardiology [14]; the American College of Cardiology Foundation/American Heart Association [39]; and the Scottish Intercollegiate Guidelines Network (SIGN) [40]. These guidelines provide a multi-continental perspective to allow for regional or cultural variations in medical management. Their selection was endorsed by the Clinical Advisory Group.

#### Reference extraction

Separate EndNote X3 libraries were set up for each of the four guidelines. The references in the bibliography of each guideline were then searched on Medline. If a bibliographic record existed it was downloaded into its corresponding EndNote library. Records without abstracts were excluded from the study as an abstract was deemed necessary for subsequent term identification.

Each record within a guideline dataset was then tagged with the name of the parent guideline before all four guideline datasets were successively merged into one dataset (n = 1297). Duplicate records were detected and deleted after manual transferral of the guideline tag from the record to be deleted to the record to be kept. This resulted in a final unique set of n = 1081 references.

**Figure 1:**
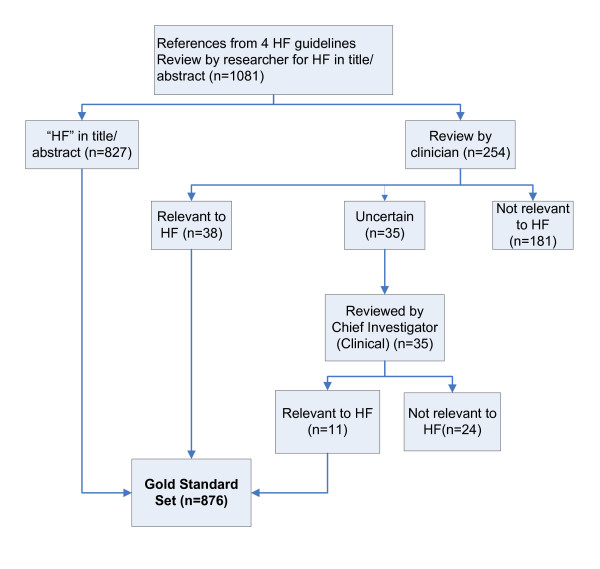
**Constructing the gold standard set**. (See attached file).

A small proportion of studies included in heart failure guidelines were identified that did not deal with a heart failure population (e.g. dyspnoea in cancer patients, depression post-myocardial infarction, or preclinical studies involving animals). These studies were eliminated from the dataset. The Research Officer (RD) scanned the titles and abstracts of all records. Any records without 'heart failure' in the title or the first sentence of the abstract were sent to a Cardiac Nurse Researcher to be checked for relevance (n = 254). The remaining references of uncertain relevance (n = 35) were sent to the study's clinical Chief Investigator (PD) for assessment. The end result was a gold standard set of n = 876 heart failure references. Figure 1 details the process of constructing the gold standard set.

#### Division of the gold standard set

The gold standard set was divided into three sets to avoid the bias inherent in validating a filter within the same set of records used to create it. These sets were: a Term Identification Set containing 10% of all references; a Filter Development Set (45%); and a Filter Validation Set (45%).

The freely available Research Randomizer program [41] was used to randomly assign 88 references (10%) from the gold standard to the Term Identification Set, which was established as a new EndNote Library. The same program was then used to randomly allocate the remaining 788 references into two EndNote library sets of 394 references each. These two distinct sets were then recreated on Medline (1950-May 2010) and saved as the 'Filter Development Set' and the 'Filter Validation Set' respectively. This was done by exporting all 394 Medline Unique Identifier numbers (UIs) in a set from EndNote into a text file; cutting and pasting the list of numbers into a search string with each number separated by the Boolean operator OR; and then running this as a search in Medline. The sets were saved using Medline's Save Search History function.

### Phase 2: Term Identification

To identify heart failure search terms using an entirely objective approach, separate frequency analyses of the controlled vocabulary terms (MeSH) and title/abstract textwords of the 88 references in the Term Identification Set were conducted. The Clinical Advisory Group was used as a cross-reference for clinical relevance. Similarly, Medline's controlled vocabulary (MeSH) thesaurus provided a useful overview of potential terms and their interrelationships.

#### Frequency analysis of Medical Subject Headings (MeSH)

Each bibliographic reference in Medline has been assigned a set of MeSH terms to describe the content and facilitate consistent retrieval [42]. In order to identify the most frequently occurring thesaurus terms describing heart failure, MeSH terms belonging to the 88 records in the Term Identification Set were exported from EndNote and saved as a list in text file format (total non-unique n = 1845). A major or minor topic focus designation is irrelevant to this study, therefore the asterisk mark (*) used to indicate a major topic focus was stripped from the start of MeSH terms. Similarly, all subheadings attached to main headings were removed in order to identify only main heading frequency. All non-MeSH terms, such as those from the Medline record's CAS Registry/EC Number/Name of Substance field, were also deleted. The edited list was then imported into Microsoft Excel and sorted alphabetically.

Before tallying the MeSH terms, the list was filtered for terms not semantically associated with the clinical manifestation of heart failure. Terms such as those describing gender, a specific study type (e.g. prospective or double-blind method), or age group (middle aged or adult) were removed. This produced a list of MeSH terms ranked by total number of appearances across all 88 records. As a MeSH term need only occur once in a Medline record for that record to be retrievable, this list was then used to determine the total number of unique records each MeSH term returned (its 'record occurrence'). It was decided *a priori *that only terms capable of retrieving 15% or more of the records in the Term Identification Set (n ≥ 13) would be considered for the filter.

#### Frequency analysis of words/phrases in title and abstract

The titles and abstracts of all records in the EndNote Term Identification Set were exported and saved as a text file. This text file was then imported into Concordance, a text analysis programme [43], which created a list of single textwords by decreasing frequency with predefined stop words omitted.

With this software it is possible to view all contexts of words appearing in the list with their frequencies. The contexts of the first 110 terms were analysed for meaningful phrases of clinical relevance to heart failure. After this, relevance and frequency dropped away sharply. A 'meaningful phrase' is defined as one which stands as an independent concept. For example, 'Left ventricular ejection fraction' is understood whereas 'Left ventricular', as an adjective, is incomplete.

This produced a weighted, ranked list of 63 single terms and phrases. Each of these were searched across the title and abstract fields of the Term Identification Set records to determine the number of unique records each retrieved (i.e. record occurrence). Again, terms and phrases selected for Phase 3 retrieved 15% or more of the records (n ≥13).

### Phase 3: Filter Development

Each candidate term was searched individually in the Filter Development Set established in Medline and ranked according to the number of records retrieved. Textwords were entered with Medline's.mp. suffix which limits the search to the title, abstract, and subject heading fields. MeSH terms were searched using Medline's.sh. suffix which forces a search of the MeSH Subject Headings field only. Textwords were not truncated and MeSH terms were searched without being focused to main topic or exploded to capture narrower, more specific concepts.

The term with the highest recall (T1) was automatically chosen for inclusion in the filter. T1 was then used as a baseline to determine the unique contribution of each of the remaining candidate terms when combined with it using the OR search operator. Terms that did not retrieve anything in addition to T1 were eliminated. The term that performed best in combination with T1 was selected for inclusion in the filter and labelled T2. The search strategy 'T1 OR T2' then became the new baseline search and all remaining terms were trialled in combination with this baseline using OR to determine the term making the next best unique contribution (T3). 'T1 OR T2 OR T3' then became the new baseline, and so on until the remaining candidate terms could no longer retrieve any additional records in the Filter Development Set.

The final search structure retrieving the maximal number of retrievals became the heart failure filter.

### Phase 4: Filter Testing

The heart failure filter developed in the Filter Development Set was run in the Medline Filter Validation Set to determine its recall in a previously unused dataset.

### Phase 5: External Validation

The performance of the filter was measured in an additional gold standard set created within Medline comprising the included and excluded studies (n = 263) from all Cochrane Heart Group heart failure systematic reviews (n = 13). These were identified by browsing the 'Topics by Cochrane Review Group' listing in the Cochrane Library. Excluded studies were considered eligible for the external validation gold standard set if exclusion was based on methodological, rather than topical, grounds. All studies needed to be indexed on Medline and contain an abstract.

### Phase 6: Post-hoc precision estimate

To get a sense of the filter's real world precision, the filter was then run in the current Medline 1950 to June Week 1 2010 file, as opposed to any gold standard set. The first 200 records retrieved were downloaded (title and abstract only) and printed. The clinical Chief Investigator (PD) then checked each record for relevance to heart failure. The post-hoc precision estimate was calculated as the number of correct inclusions as a proportion of the set of 200.

## Results

The gold standard set of n = 876 heart failure references spanned a total number of 128 unique journal titles and the years 1976 to 2008. Journal titles reflected the interests of 44 distinct areas within health care, based on the National Library of Medicine's classification system for describing a journal's overall scope [44]. Areas include: cardiology, general medicine, pulmonary medicine, nephrology, vascular disease, drug therapy, psychiatry, nursing, health services, surgery, oncology, sports medicine, rehabilitation, transplantation, complementary therapies and geriatrics.

### Term Identification

Analysis revealed that *Heart failure *(MeSH) and *heart failure *(textword) had the highest record occurrences (62/88, 70.4%; 77/88, 87.5%) in the Term Identification Set (n = 88).

When the pre-established cut-off point for potential term inclusion in the heart failure filter was applied, two MeSH terms and seven textwords qualified: *Heart failure *and *Ventricular dysfunction, Left *(MeSH terms); and *heart failure, CHF, congestive heart failure, left ventricular ejection fraction, chronic heart failure*, *New York Heart Association*, and *cardiomyopathy *(textwords).

Candidate MeSH terms and textwords and their frequencies are reported in Table 1.

**Table 1:** Most frequent terms identified in the Term Identification Set

Terms	Record Occurrence	% Record Occurrence
**MeSH**		
Heart failure	62	70.5%
Ventricular dysfunction, Left	14	15.9%
**Textwords/phrases**		
Heart failure	77	87.5%
Congestive heart failure	36	40.9%
New York Heart Association	24	27.3%
Left ventricular ejection fraction	23	26.1%
CHF	20	22.7%
Chronic heart failure	17	19.3%
Cardiomyopathy	15	17.1%

### Filter Development

With a recall of 92.6%, the individual term which best performed with respect to retrieving articles in the Filter Development Set was *heart failure.mp*. (This constitutes both a textword and MeSH search.) The details of subsequent search permutations and their performance are reported in Table 2. The final best performing search (the heart failure filter) was the four-term search: '*heart failure.mp. OR Ventricular dysfunction, Left.sh. OR cardiomyopathy.mp. OR left ventricular ejection fraction.mp*.' (recall = 96.9%).

**Table 2:** Search permutations and performance (recall) in the Filter Development Set

Baseline search	Representation	No. records retrieved in Filter Development Set (n = 394)	Recall (%)
Heart failure.mp.	T1	365	92.6
	T1 OR left ventricular ejection fraction.mp.	372	94.4
	T1 OR cardiomyopathy.mp.	373	94.7
	T1 OR Ventricular dysfunction, Left.sh.	373	94.7
Heart failure.mp. OR cardiomyopathy.mp.	T1 OR T2(a)*	373	94.7
	T1 OR T2(a) OR left ventricular ejection fraction.mp.	378	95.9
	T1 OR T2(a) OR Ventricular dysfunction, Left.sh.	380	96.4
Heart failure.mp. OR Ventricular dysfunction, Left.sh.	T1 OR T2(b)*	373	94.7
	T1 OR T2(b) OR left ventricular ejection fraction.mp.	376	95.4
	T1 OR T2(b) OR cardiomyopathy.mp.	380	96.4
Heart failure.mp. OR Ventricular dysfunction, Left.sh. OR cardiomyopathy.mp.	T1 OR T2(b) OR T2(a)	380	96.4
	T1 OR T2(b) OR T2(a) OR left ventricular ejection fraction.mp.	382	96.9

*The two terms *cardiomyopathy.mp*. and *Ventricular dysfunction, Left *both qualified for term 2 (T2), having retrieved the same number of references in combination with T1. They have been labelled T2(a) and T2(b) in order to assess their relative performance as part of the baseline search.

Note: The OvidSP Medline suffix '.mp.' forces a search on the title, abstract, subject headings, and chemical registry fields while the '.sh.' suffix forces a search on the subject headings field alone.

The textwords *CHF, congestive heart failure, chronic heart failure *and *New York Heart Association *did not retrieve any additional records when combined with *heart failure.mp*. In other words, these terms only ever appear in association with *heart failure *in the filter development set, not independently. They were therefore excluded from the final filter.

### Filter Testing

The final four-term heart failure filter (Table 3) was then run in the Filter Validation Set, retrieving 387/394 records (recall = 98.2%). The 7 records not retrieved were indexed with the MeSH terms *Pulmonary edema *(n = 3), *Coronary disease *(n = 1), and both *Heart transplantation *(n = 1) and *Implantable defibrillators *(n = 2) without reference to an underlying condition.

**Table 3:** The final OvidSP Medline heart failure filter

#	Searches
1	heart failure.mp.
2	ventricular dysfunction, left.sh.
3	cardiomyopathy.mp.
4	left ventricular ejection fraction.mp.
5	Or/1-4

Note: The OvidSP Medline suffix '.mp.' forces a search on the title, abstract, subject headings, and chemical registry fields while the '.sh.' suffix forces a search on the subject headings field alone.

### External validation

The heart failure filter was then validated in an alternative gold standard set comprising n = 269 references from n = 13 Cochrane Heart Group systematic reviews of heart failure interventions. The filter retrieved 263/269 records (recall = 97.8%). The 6 records not retrieved were indexed with the MeSH terms *Pulmonary edema *(n = 1), *Edema *(n = 2), *Chronic diseases *(n = 1), *Cardiovascular diseases *(n = 1) and *Heart diseases *(n = 1).

### The post-hoc precision estimate

The heart failure filter was run in Medline, outside the gold standard set. The first 200 records retrieved were analysed for relevance to heart failure. Out of the 200 records, 150 were considered correct inclusions. The filter therefore achieved a post-hoc precision estimate of 75%. The irrelevant retrievals were analysed to determine if a specific term or terms were overrepresented in this set. Over half of the irrelevant references possessed 'heart failure' either in the title or abstract (n = 17/50) or as a MeSH term (n = 9/50). The filter textword *cardiomyopathy *and MeSH term *Ventricular dysfunction, Left *contributed equally to the irrelevant set (n = 11 each) and *left ventricular ejection fraction *brought in only two unique irrelevant references. Subheadings occurring in combination with filter terms in the MeSH field (e.g. Heart failure/drug therapy) were also examined. Although the subheadings 'etiology', 'physiopathology' and 'ultrasonography' predominate in the irrelevant set, the very same combinations occur in the relevant set.

## Discussion

This study shows that it is possible to use an efficient and objective methodology to create a high recall heart failure filter capable of retrieving approximately 98% of relevant records in two validation sets. This suggests that the risk of the filter missing an important study is minimal. Given that it was not possible to measure specificity or precision within our gold standard set, a post-hoc estimation of precision was used to gauge the filter's performance under 'real world' conditions. It was anticipated that the filter's high recall could come at some cost to precision once it was run across the full Medline database, however, in a set of 200 retrievals, the filter achieved an estimated precision of 75%. In other words, our reviewer encountered one possibly irrelevant reference for every three relevant ones. As a point of comparison, this precision estimate exceeds precision percentages calculated for 38 search filters designed to retrieve randomised controlled trials in Medline. Precision for these filters ranged from 1.2% to 56.4% [25].

Over half the irrelevant references possessed 'heart failure' as either a textword or a MeSH term, and both *cardiomyopathy *and the MeSH term *Ventricular dysfunction, Left *contributed equally to the irrelevant set. These findings indicate that an improvement in the filter's precision would not be a simple matter of removing an overly sensitive term. In fact, no single filter term, other than the indispensible *heart failure*, made a major contribution to the irrelevant set. The removal of any term other than *heart failure *would therefore have a significant effect on filter recall while potentially improving precision only in the smallest degree. Indeed, the irrelevant references could best be designated 'irrelevant relevancies'. They were rejected because notions of relevance and utility did not connect on this specific occasion for a specific reviewer [45]. The participation of a second reviewer may have moderated this effect.

Search filter development exposes complexities and controversies surrounding concepts and definitions. It was clear at the outset of this study that the single search term *heart failure *captures the majority of articles but does not capture the entire corpus of literature. For example, a significant number of references across the full gold standard set were not indexed with the *heart failure *MeSH despite *heart failure *appearing in the title and abstract. These references were indexed with the MeSH terms *Ventricular dysfunction, Left*; *Cardiomyopathy, Dilated*; or *Cardiac output, Low*. In fact, heart failure is an understandably difficult concept to index by virtue of being a heterogeneous syndrome with many potential aetiologies, diverse clinical features, numerous clinical subsets, and no universally agreed upon definition [16]. When used in association with 'heart failure' *chronic *can mean 'congestive' while *acute *can mean 'time from diagnosis', 'severity', or 'decompensated chronic heart failure' [14]. Two of the terms in the filter, 'left ventricular dysfunction' and 'cardiomyopathy', are high performance heart failure search terms but would not be considered synonyms for heart failure as a symptomatic condition. Similarly, 'left ventricular ejection fraction' is a measurement in cardiac imaging commonly used to describe the underlying cardiac status of a heart failure patient and does not constitute a medical condition. These terms, however, appear to be reliably indicative of the heart failure population.

In view of the complexities surrounding heart failure terminology, it was important that filter term selection was based on an objective approach. An *a priori *decision was made to not automatically truncate textwords to capture all word ending variations (e.g. cardiomyopath*) as this could increase the filter's recall to the detriment of precision. Similarly, MeSH terms in the filter were not exploded as a matter of course to retrieve on their narrower, more specific concepts. In the case of *heart failure*, this meant omitting the related, narrower concepts *Edema, Cardiac *and *Dyspnea, Paroxysmal*. Ultimately, this was a justified omission as these terms did not even appear in the ranked frequency list.

Although an objective approach to filter term derivation was stringently adhered to, several relatively subjective decisions may have indirectly affected the final filter outcome. One was in the choice of term frequency cut-off level (n ≥13) which dictated the terms that went on to become shortlisted candidates. The cut-off level may have eliminated infrequently occurring terms with high discriminatory capacity. Another decision relates to the sample size of the Term Identification Set. The filter was derived from 88 references which may or may not be an adequate number in terms of power. To date, there is little clarity around the issue of sample size in the literature [31].

Similarly, topic filter methodology is not yet mature enough to make any conclusive statement on the potential biasing effect of either including or excluding references without abstracts from the gold standard and, hence, the frequency analysis process. This study eliminated references without abstracts as it was clear, in the majority of cases, that relevance to heart failure could not be determined on the basis of title alone. An assessment of relevance would require obtaining the full-text article or relying solely on indexer-assigned MeSH terms. Furthermore, the value of frequency analysis lies in its ability to elucidate the less obvious and, potentially, more highly sensitive terms and it was expected that these terms would occur in abstracts rather than titles. The phrase 'left ventricular ejection fraction' is one example of a phrase not expected to occur frequently in heart failure article titles. However, in recognition of the potential significance of this decision, this study ran the filter within the excluded set of articles without abstracts (n = 168) post-hoc. The filter failed to retrieve 36% of this set (n = 60). An analysis of the MeSH associated with the non-retrieved references revealed that only 7 were questionable exclusions as they included terms such as *Heart transplantation *and *Cardiac output, Low*. The remaining references were not picked up by the filter as they were indexed with the more general terms *Cardiology*, *Cardiovascular diseases *or *Coronary disease*, or non-heart failure terms such as *Myocardial infarction, Atrial fibrillation, Gout*, and *Metabolic syndrome X*. In this instance, there was limited bias associated with the decision to exclude articles without abstracts. Nevertheless, this example is illustrative of the complexity of filter development and further study is warranted to test different aspects of underlying methodology such as this.

The representativeness of a gold standard created by extracting studies from a systematic review or guideline is inexorably linked to the quality and comprehensiveness of the search strategies employed to identify these studies in the first place. In other words, a review or guideline based on a limited search using a few search terms will, in turn, produce a gold standard of limited scope. A filter derived from such a gold standard would indirectly reflect the original limited search and lack generalisability. Using multiple guidelines that explicitly describe the methods used to locate the evidence in the first place and which employ a variety of search strategies would work towards mitigating this potentially biasing effect.

The four guidelines in this study were selected on the basis of their scope, currency and their stated evidence-based approach to the literature. If guideline scope is wide enough, it should be possible to create a filter capable of capturing the evidence at all points in a condition's natural history or trajectory. If the articles within the guidelines represent a range of journal titles and publication years, as ours did, the filter derived from them can be considered to have generalisability to the wider literature and the ability to account for changes in terminology across time. Ideally the gold standard set relevance check should be performed by two independent clinicians, but the use of only one such reviewer was based on resource constraints in our study. This may have resulted in some relevant references being omitted and some irrelevant ones being retained, potentially introducing a degree of bias.

The articles in clinical practice guidelines reflect a range of study designs from high quality systematic reviews and randomised controlled trials of therapeutic interventions, to designs which may not have the same level of rigour but are the only available evidence on which to base a clinical recommendation. The heart failure filter performed well in retrieving across all research designs in the Filter Validation Set. Notably, the filter also performed well in a gold standard based on a single research design as it had a high level of recall in the Cochrane Validation Set comprising randomised controlled trials extracted from Cochrane systematic reviews.

This study shows that clinical practice guidelines are an alternative source of references to the traditional journal hand search when constructing a gold standard for search filter development. Guidelines may even prove to be a superior source if the relevance of retrievals is prized above recall. This is due to the fact that guidelines, by their nature, focus on the clinical knowledge considered essential in a particular field of practice. This focus, combined with a broad scope and non-reliance on one study type alone, also differentiates the literature at the core of guidelines from that of systematic reviews.

While there are already multiple published studies on methodological filter development that report using systematic reviews to create a gold standard, the relative appropriateness of such reviews for topic-based filters has not yet been quantified. Additional research that compares the performance of a topic filter built on a guideline gold standard with one built on a systematic review gold standard may represent a potential future research direction.

Finally, the guideline approach potentially offers filter developers substantial savings in terms of time and labour. In particular, it obviates the need for a dual review of a large number of full-text articles in order to determine relevance, a process which has been the hallmark of the traditional hand search approach. While guideline citations still need to be assessed for relevance to the topic of the filter, the prevalence of relevant citations in guideline bibliographies and their evident context make this a much simpler process. A cost-benefit analysis comparing both approaches could make a useful and timely contribution to the field of filter development.

## Conclusion

We developed a topic-based filter with high recall and an estimated precision of 75% for retrieving studies on heart failure in Medline. In doing so, we trialled an alternative and entirely feasible methodology for creating a gold standard set based on multiple clinical practice guidelines rather than traditional hand searching or systematic reviews. A validated filter is now available to support health professionals seeking to retrieve the literature relevant to heart failure.

## Competing interests

The authors declare that they have no competing interests.

## Authors' contributions

JT conceptualised and designed the study, established the Clinical Advisory Group, supervised data analyses and the interpretation of findings, and reviewed several manuscript drafts. RD contributed to the development of the methodology, conducted the study and drafted the manuscript. RS contributed to the development of the methodology, provided advice throughout the conduct of the research, and edited and revised several manuscript drafts. PD provided clinical oversight, coordinated the work of the Clinical Advisory Group, evaluated the retrieved references for clinical relevance in the course of the post-hoc precision analysis, and reviewed several manuscript drafts. All authors read and approved the final manuscript.

## Funding

The CareSearch project undertook this work in acknowledgement of the end-of-life care needs of heart failure patients and as part of its role to support health professionals providing palliative care across all settings.

The CareSearch project is funded by the Australian Government Department of Health and Ageing.

## Pre-publication history

The pre-publication history for this paper can be accessed here:

http://www.biomedcentral.com/1471-2288/11/12/prepub
